# Qualitative exploration of cultural factors influencing diet among African-, Caribbean- and US-born Blacks living in the northeast USA

**DOI:** 10.1017/jns.2019.19

**Published:** 2019-07-16

**Authors:** A. G. M. Brown, R. F. Houser, J. Mattei, A. H. Lichtenstein, S. C. Folta

**Affiliations:** 1 National Heart, Lung, and Blood Institute, National Institutes of Health, 6701 Rockledge Drive, Bethesda, MA 20892, USA; 2 Department of Nutrition, Harvard T.H. Chan School of Public Health, 665 Huntington Avenue, Building 2, Boston, MA 02115, USA; 3 Tufts University Friedman School of Nutrition Science and Policy, 75 Kneeland Street, Boston, MA, USA; 4 Tufts University, Jean Mayer USDA Human Nutrition Research Center on Aging, 711 Washington Street, Boston, MA 02111, USA

**Keywords:** Culture, Ethnicity, US Black population, African-ancestry populations, Caribbean-born population, Diet, Dietary acculturation

## Abstract

Limited research considers the ethnic and cultural diversity among the US Black population, and how this diversity influences diet. The purpose of the present qualitative study is to (1) explore the influence of culture, nativity and ethnicity on the diet of US-born, African-born and Caribbean/Latin American-born Blacks and (2) explore a model of dietary acculturation among the African-born and Caribbean/Latin American-born Blacks. The purposive sample included twenty-two US-born, fifteen Caribbean/Latin American-born and ten African-born Blacks (*n* 47) living in Boston, who participated in either an in-depth interview (*n* 12) or a focus group (five groups, size 5–9). Satia-Abouta's model of dietary acculturation informed the interview and focus group questions, which explored the influence of psychosocial factors, taste preferences and environmental factors on dietary changes. NVivo 10 software was utilised for the coding and analysis. Topics based on *a priori* and *posteriori* analyses included differences in psychosocial factors and taste preferences and environmental factors by nativity. Caribbean/Latin American-born and African-born Blacks expressed the importance of cultural identity in their dietary preferences and found adaptive strategies to maintain cultural diet, while US-born Blacks demonstrated a variety of preferences for traditionally African American foods. Environmental factors varied by place of birth and residence, with US-born Blacks citing poorer quality and limited affordability of foods. These findings suggest the importance of psychosocial and environmental factors in shaping the diet of the ethnically diverse US Black population and underscore the dietary diversity within and across the different ethnic groups of Blacks.

Blacks in the USA are generally reported to have poor diet quality and to not meet dietary recommendations^(1–4)^. For example, studies have shown that intake of vegetables, whole grains, milk, dietary fibre, K and Ca is lower among Blacks than Whites^(1–5)^. To add to these findings, future research could account for the ethnic diversity among the US Black population, and how this diversity may reflect the intra-ethnic variations of dietary patterns. While some Blacks have been in the USA for many generations, others are recent immigrants from Africa, the Caribbean or Latin America, and first- and second-generation descendants. The Black immigrant population has grown significantly since the 1960s, with Caribbean-born and African-born self-identified Black immigrants making up an estimated 8·7 % of the US Black population, and is projected to increase to 16·5 % by 2060^(6,7)^. This heterogeneity among Blacks includes cultures and social realities that potentially influence food and taste preferences and dietary patterns. Understanding this diversity could inform more effective nutrition interventions, dietary recommendations and national data collection.

Few studies have explored dietary diversity among Blacks living in the USA^(8–12)^, whereas several studies have explored the diets of British Afro-Caribbean and West African populations in the UK^(9,10,13)^. One such study examined the diets of Afro-Caribbean individuals and concluded that dietary modification suggestions for diet-related diseases should consider cultural contexts because Caribbean countries have unique food habits and dietary patterns that persist in first and later generations after immigration^(9)^. An ethnographic study exploring the relationship between migration, foodways, ethnic identities and gender among Ghanaians in London found practices representing the hybrid identities of Ghanaians (i.e. turkey and trimmings for Christmas and traditional Sunday roast)^(13)^. In the USA, one study compared the diet of African Americans and Haitian Americans with and without type 2 diabetes, showing that Haitian Americans had significantly better diet quality with greater intakes of fruit, vegetables and whole grains than the African American study participants^(12)^. A qualitative study comparing the diets of Afro-Caribbean and African American women indicated cultural variations in traditions of food and food preparation between the two groups, underscoring the importance of not using a one-size-fits-all approach for community interventions^(8)^.

Related to the cultural influences of diet is the process of acculturation and changes in habits of immigrant groups. Acculturation is a gradual process that occurs over time. Factors such as length of residency, age of migration, sex, socio-economic status, reasons for migration, place of settlement, household make up and social and physical environments all play a role in the process of acculturation^(14)^. To date, most of the research related to dietary acculturation has been conducted among Hispanic and Asian immigrant groups, for which migration and acculturation have been shown to be associated with changes in lifestyle behaviours and dietary composition with increased length of residency^(15–19)^. While various conceptual models have been proposed to explain the general process of acculturation^(14)^, the Satia-Abouta model of dietary acculturation (Fig. 1) specifically posits several key contributors to changes in diet among immigrant groups, including socio-economic (i.e. education level, household income), demographic (i.e. sex, length of time in the USA, age of migration) and cultural factors^(20)^. These factors are thought to predict the process of change in psychosocial factors, such as attitudes and beliefs about food, taste preferences and food availability and preparation^(20)^.

**Fig. 1.**
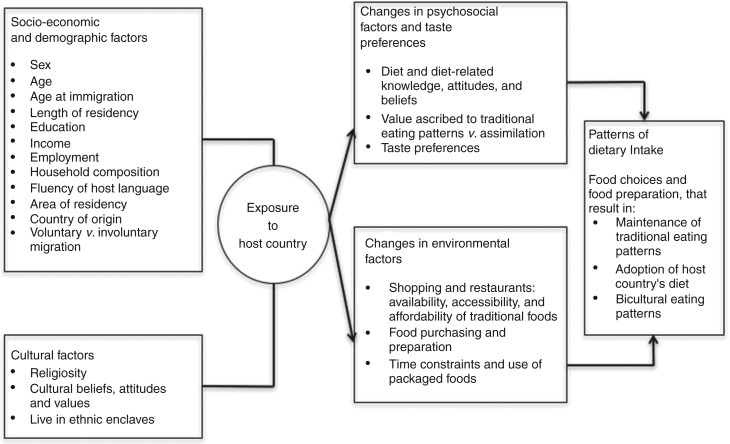
Model of dietary acculturation adapted from Satia-Abouta's model^(20)^.

Given the applicability of the model of dietary acculturation and the limited research among this demographic^(15,16,18)^, we designed a qualitative study with the goal of (1) exploring the influence of culture and ethnicity on the diet of an ethnically diverse Black population living in Boston, MA, USA, specifically US-born, African-born and Caribbean/Latin American-born Blacks and (2) exploring a model of dietary acculturation among the study sample. Using this model, we examined the potential influence of changes in environmental and psychosocial factors in shaping the diet of ethnically diverse Blacks living in the USA.

## Methods

This qualitative study used in-depth interviews and focus groups with US-born, African-born and Caribbean/Latin American-born Blacks living in Boston, MA, USA to examine factors within and across groups with respects to the model of dietary acculturation. Specifically, we conducted four in-depth interviews for each ethnic heritage, two focus groups for US-born and Caribbean-born Blacks and one focus group for African-born participants (details outlined in the Results section). We developed a semi-structured discussion guide (used for both the in-depth interviews and focus groups), informed by the Satia-Abouta model of dietary acculturation.

We structured the discussion guides into two main topic areas from the Satia-Abouta model (Fig. 1) including psychosocial factors and taste preferences, and environmental influences (Table 1). Questions relating to the psychosocial constructs explored how the participants felt their culture and social networks influenced the way that they ate, their preferences for cultural foods, specific cultural food traditions that they maintain and any changes in their preferences. In the environmental domain, we asked participants about the availability and affordability of culturally appropriate foods in their community, the impact of their social environments and socio-economic influences. The interview and focus group questions were slightly different between non-US-born and US-born participants. Specifically, any questions pertaining to changes in diet since migrating to the USA or circumstances of moving to the USA and how this may have influenced diet were not applicable to the US-born participants (see specific questions in Table 1). This methodology allowed us to use the model of dietary acculturation to examine participants' perceptions of the factors that influence their diet and, for non-US-born participants, any dietary changes since immigrating to the USA. Upon review and revision by the study team, we pilot-tested the discussion guide with one focus group and one in-depth interview and refined to improve clarity and flow.

**Table 1.** Major topics from Satia-Abouta's model, discussion guide and questions^(20)^

Topic	Questions
Environmental factors	In what way has living in Boston influenced your eating of traditional foods from your culture?
	Describe the availability of traditional/culturally appropriate foods in your community.
	If traditional/culturally appropriate foods are available, how would you describe their prices? Affordable? Not affordable?
	If traditional/culturally appropriate foods are available, describe your travel time to get these foods.
	Describe the time that you have to devote to preparing foods from your home country or culture.
	Describe the availability of restaurants that serve the food or similar food from your home country.
	In what way does time availability play a role in your preparation of foods from your home country?
	Do you think your education or income had an impact on the way that you ate when you first came to this country?* Has this changed at all today? And if so, how?
	In what ways do you think the other people who currently live with you influence your eating? (For example, older adults in the household)
Psychosocial factors and taste preferences	In what ways is your preference for foods influenced by your culture?
	In what ways has your preference for foods changed (if at all) since you moved to the U.S.?*
	In what ways has your beliefs about diet-related disease such as heart disease, diabetes and obesity changed since you moved to the U.S.?*
	How have these beliefs influenced the way you eat?
	What are your thoughts on African Americans’ (or people in your country of birth's) perception about diseases such as high blood pressure, diabetes (sugar) and heart disease?
	How have these beliefs influenced the way you eat?
	How does the culture of your ethnic group influence the way you eat?
	Can you share any particular foods/dishes that you eat from your culture?
	In what ways has your diet changed since you've moved to the U.S.?*
	In what ways has your diet stayed the same since you've moved to the U.S.?*
	In what ways do you think your age of migration might have influences on your diet today?*
	In what ways did your personal circumstances of moving to the U.S. (i.e. more educational opportunities and for other economic opportunities) contribute to your diet when you came to the U.S.?*

U.S., United States of America.

* Question asked to Caribbean/Latin American-born and African-born Blacks.

Tufts University Health Sciences Institutional Review Board approved all study procedures. We used purposive sampling by the placement of promotional flyers and on-site recruitment at community-based organisations and on-site tabling at community events in predominately Black/African American/Afro-Caribbean neighbourhoods in Boston over a 3-month period. The research manager recruited and screened potential participants who expressed initial interest in the research study by telephone or in-person. According to the Satia-Abouta model, demographic factors such as age, sex, age of migration, number of years in the USA and household composition are particularly important factors to consider in the dietary acculturation process. Given this host of factors, to help focus this study, our sample was restricted to adults between the ages of 40 and 70 years without children in the household. Inclusion criteria were therefore self-identifying as Black/African American; aged 40–70 years; English-speaking; being born in either the USA, countries throughout the Caribbean/Latin America, or countries throughout Africa. Exclusion criteria included having children under the age of 18 years living in the household, given the influence of children on foods served in the household^(21)^. Our target population focused on middle-aged adults aged 40–70 years given the increased risk for diet-related diseases in this demographic. Initial targets for recruitment included twenty US-born Blacks, twenty Caribbean/Latin American-born Blacks and twenty African-born Blacks to obtain equal representation from each region of birth. We designed the purposive sample to provide a range of perspectives given the large geographic regions while also providing enough data to reach saturation on broader themes related to the acculturation model. We determined saturation when no new themes emerged with additional data^(22)^. It was not intended to provide data on detailed differences in dietary intake among individual countries and cultures represented. We randomly assigned each participant to participate in either a 60-min in-depth interview by telephone or a 90-min focus group. All participants received a $50 Visa gift card for participation. Prior to the in-depth interview or focus group, each participant read an informed consent agreement form and completed a brief questionnaire, which included questions related to sociodemographic factors, place of birth, year and age of migration and cooking and eating habits. For the in-depth interviews, participants completed the questionnaires via the online Qualtrics survey software. All focus groups and in-depth interviews were conducted by the lead study investigator (A. G. M. B.), who was trained in qualitative methodology and research approaches through the Boston Nutrition and Obesity Research Center. Focus groups were conducted at community-based organisations such as churches and libraries in predominately Black/African American neighbourhoods in Boston.

All in-depth interviews and focus groups were digitally recorded and preliminary transcription was conducted using online transcription software, speechmatics (https://www.speechmatics.com)^(23)^. All data were de-identified to ensure anonymity of study participants and maintain their confidentiality. Graduate research assistants then manually reviewed and finalised the transcripts. We analysed the data using thematic analysis^(24)^. First, the team developed the initial codebook based on emergent themes from the transcripts. We then revised the codebook based on coding an initial transcript and discussion among study team members. Inter-coder reliability was then established by randomly selecting one transcript and comparing codes between the lead analyst (A. G. M. B.) and the graduate research assistants. For any codes that failed to achieve 80 % agreement or better, we met to discuss discrepancies and further refined the codebook to clarify code definitions. NVivo 10 software was utilised to assist in the coding and analysis process (QSR International)^(25)^. To analyse the data, we conducted matrix coding queries based on characteristics, such as region of birth, education, income, and, among foreign-born participants, length of residency and age at arrival. Based on patterns and counts within the matrix coding queries, we identified themes that emerged from the data.

## Results

### Sample characteristics

Demographic characteristics of the sample by region of birth are summarised in Table 2. The sample (*n* 47) included twenty-two US-born Blacks, fifteen Caribbean/Latin American-born Blacks and ten African-born Blacks. Initial target numbers for the study sample were not met due to recruitment challenges and time constraints of interested participants. For the US-born study participants, we facilitated two focus groups (*n* 9 each) and conducted four telephone interviews. For the Caribbean/Latin American-born participants from Honduras, Jamaica, Barbados, Trinidad and Tobago and Puerto Rico, we held two focus groups (*n* 5, *n* 6), and conducted four interviews. For the African-born participants from Somalia, Ethiopia, Cape Verde and Nigeria, we held one focus group (*n* 6) and conducted four interviews. Among the US-born Black participants, 13·6 % (*n* 3) were considered first generation (parent(s) born outside of the USA) and one participant was considered second generation (grandparent(s) born outside of the USA).

**Table 2.** Sociodemographic characteristics and food-related behaviours of ethnically diverse US Blacks living in the northeast USA (Numbers and percentages; mean values with their standard errors; mean values and standard deviations)

	US-born (*n* 22)		Caribbean/Latin American-born (*n* 15)		African-born (*n* 10)	
	*n*	%	*n*	%	*n*	%
Age (years)						
Mean	57·6		55·5		56·8	
se	1·6		2·1		2·4	
Female	15	68·2	12	80·0	4	40·0
Educational attainment						
High school or less	11	50·0	4	26·7	1	10·0
Some college	5	22·7	3	20·0	2	20·0
≥College degree	6	27·3	8	53·3	7	70·0
Marital status						
Never married	13	59·1	11	73·3	6	60·0
Married	2	9·1	0	0·0	1	10·0
Divorced, separated, widowed	7	31·8	4	26·7	3	30·0
Annual income						
<$24 999	14	63·6	1	6·7	2	20·0
$25 000–$49 999	4	18·2	5	33·3	3	30·0
≥$50 000	3	13·6	5	33·3	5	50·0
Prefer not to reply	1	4·6	4	26·7	0	0·0
Length of residency	–					
<10 years	–		1	6·7	1	10·0
10–29 years	–		1	6·7	3	30·0
≥30 years	–		13	86·7	6	60·0
Age of migration (years)	–					
Mean	–		19·0		28·2	
sd	–		3·9		3·1	
Age of migration						
Younger than 18 years	–		9	60·0	1	10·0
Older than 18 years	–		6	40·0	9	90·0
Reasons for migration*	–					
Education	–		6	40·0	4	40·0
Work/employment	–		5	33·3	10	66·7
Unite with family	–		10	66·7	1	10·0
Conflict/natural disaster in home country	–		0	0·0	3	30·0
Other	–		4	26·7	2	20·0
Cooking responsibilities						
Mainly responsible	16	72·7	11	73·3	8	80·0
Shared responsibility or someone else's responsibility	6	27·3	4	26·7	2	20·0
Grocery shopping responsibilities						
Mainly responsible	18	81·8	12	80·0	7	70·0
Shared responsibility or someone else's responsibility	4	18·2	3	20·0	3	30·0
Food intake	–					
Mainly food from my country of birth	–		1	6·7	2	20·0
Mostly food from my country of birth and some American food	–		3	20·0	3	30·0
Equal amounts of both food from my country of birth and American foods	–		9	60·0	4	40·0
Mostly American foods and some food from my country of birth	–		2	13·3	1	10·0

* Participants had the option to choose more than one reason for migration.

The mean age was similar for US-, Caribbean/Latin American- and African-born (57·6 (sd 1·6) years, 55·5 (sd 2·1) years and 56·8 (sd 2·4) years, respectively). The mean age for migration for Caribbean/Latin American-born and African-born participants was 19·0 (sd 3·9) years and 28·2 (sd 3·1) years, respectively. A greater percentage of the Caribbean/Latin American-born participants were female (80 %) compared with the African-born (40 %) and US-born (68·2 %) participants. Caribbean/Latin American-born and African-born Blacks were more likely to have a college degree or higher, 53·3 and 70·0 %, respectively, in comparison with 27·3 % of their US-born counterparts. Similarly, compared with US-born Blacks, foreign-born Blacks were more likely to have a family income of ≥$50 000.

Among the foreign-born study participants, most of both the Caribbean/Latin American-born (86·7 %) and African-born (60·0 %) study participants had lived in the USA for ≥30 years. The average age of migration was 19·0 years for Caribbean/Latin America-born and 28·2 years for African-born Blacks. Of the Caribbean-born, 60·0 % migrated to the USA before they were 18 years, compared with only 10 % of the African-born sample. Reasons for migration varied between region of birth, with most Caribbean/Latin America-born seeking education or family reunification and most African-born migrating in search of education and employment opportunities.

In terms of eating patterns, most Caribbean/Latin America-born (60·0 %) and African-born (40·0 %) Blacks indicated eating equal amounts of American food and food from their country of birth. Most indicated being mainly responsible for the cooking and grocery shopping in their household, regardless of country of origin.

### Qualitative data

Several *posteriori* themes emerged within the *a priori* question topic areas that were based on the Satia-Abouta model. Specifically, psychosocial factors and taste preferences varied by place of birth and environmental factors played more of a role in shaping the diets of US-born Blacks over Black immigrant groups. All emergent themes with corresponding representative quotes are presented in Table 3. Because themes did not differ by the methodology, they are presented together.

**Table 3.** Emergent themes, summary of qualitative data and representative quotes regarding cultural aspects of diet among ethnically diverse US Blacks living in the northeast USA

	US-born	Caribbean/Latin American-born	African-born
Changes in psychosocial factors and taste preference			
Value, preference and influence of cultural foods Strong preference for cultural foods among Caribbean-born and African-born Blacks Mixed preference for US-born Blacks, with influence of value for health	‘I don't know. I like the collard greens and the greens and the peas and the way they definitely do eat their vegetables. But you know I've been trying to minus the pork and trying to find alternatives. But I like that they are very strong about eating their vegetables. I love the way they season their meats. And I love love the way they barbecue.' (in-depth interview) ‘I'm getting tired of African American food myself because I've eaten so much.’ (focus group)	‘I still prefer Honduran food over anything.’ (focus group) ‘So I think that it sort of set it in stone what I would like to eat….Those things become much more important to me so that even going forward I always try to keep an element of that ethnic food and traditional you know kitchen cooking.’ (in-depth interview)	‘Well I can't live without it, I have to have it so it's important because I grew up with it. Also you just get used to it. I cannot not eat it at least for a couple of days. So it has value, it's important, that's how I identify.' (in-depth interview) ‘Food is so important in our culture. Like I said I just went back and I got to the point I had to tell my family, I'm tired of eating (laughter). Because food is the way we show appreciation, food is the way we show our love. And I felt bad.’ (in-depth interview)
Differences in food preferences based on region of birth In comparison with US-born participants, African-born and Caribbean/Latin-born Blacks expressed eating more fish, fruit, vegetables, and using more spices and seasonings as well as variety of these foods	‘ …I believe the food I myself was brought up on was fried chicken. Chicken. Bone. I love chicken bone. I know where it comes from. And pork and all that. I'm used to eating fried food is my biggest problem. I don't bake notin. I fry it.’ (focus group)	‘Well I think it looks like breakfast for us. And it's actually fresh stuff. It's spinach and green bananas and ackee, stuff like that. I guess it can be heavy but I feel like it's healthy, it's from the earth. It's bananas, it's potatoes, it's not like eating French toast with syrup poured on top of it. Not to say there's anything wrong with that, but on Sundays you'll make green bananas, spinach with toast or eggs or whatever. To kind of switch it up a little bit.' (focus group) ‘I would use more curry and turmeric, and I make my own seasoning like with onions and garlic and peppers. A mixture of sweet marjoram and all that kind of stuff together and make own seasoning, so I would use too much of the salty stuff.' (focus group)	‘But mostly from my part of Nigeria, we ate mostly fish, mostly fresh fish and some smoked. But you don't find the smoked fish that is smoked like the way we smoke it in Nigeria…So fish is our major, in fact, I make it a point to make sure we eat fish at least twice a week. And beef we still eat, not sparingly but more fish than beef and more chicken than beef too.’ (in-depth interview) ‘Vegetables are the same as here. We eat spinach, we eat greens all green leaves, we eat carrots, potatoes. Cauliflower. All kinds of vegetables, we have them back home but like I said it ate limited when I was back home but here my portion has gone up.’ (in-depth interview)
Adaptive strategies Change in ingredients based on resources Bulk meal preparation Change in preparation methods and ingredients for health reasons Portion size Home country travel	‘I still eat some of the stuff I was grown upon now. My mother cooked a lot of chitterlings and pig feet, stuff like that. I don't eat them anymore…Today I am trying practising eating more healthy…I'm trying to change my eating habits, not eat a lot of fried food. Try to eat more baked. Eat a little less.’ (focus group) ‘Because my mother came from the south, and there were certain foods she wouldn't allow in her house. We didn't eat pork. She wouldn't allow it because over time…her education influenced the type of food we ate in the house…The same thing applies to me as well with certain foods. Like collard greens, I'm going to put some smoked turkey in there to make it more healthy…because all that stuff can pretty much clog your arteries…’ (focus group)	‘We still eat the same cultural food, but we might just cut back on the portion…We don't eat much – Some things we don't get, but what we get here, we eat it. Just cut back the portions. The fish, the salt fish, the callalou. We will cook it probably occasionally, but we just cut back on portion. Not the big plate we have in front of us (laughs). The pig's tail, we eat still.' (focus group) ‘We try not to do the Crisco oil anymore. We only use it in frying, but we would use the olive oil more.' (focus group) ‘Yes I do get mauby if I go down to the Caribbean, I get it in a syrup form and I would bring that back and I would make my own.’ (in-depth interview)	‘It's now at home, so I choose to cook outside in the garage because it's a lot of onion, so smelly, so spicy, so I use Saturday, I cook Eritrean or Ethiopian food and then save it for a week. So in between Thursday and Friday we can have any Ethiopian dish like pasta.’ (focus group) ‘But it's so hard to find the teff…So you mix it up. Quarter of teff, quarter of barley and a little rice and you mix it up and make it as injera.’ (focus group) ‘So, you see, even the substitutes we were using are getting off our radar, for one reason or the other, we don't need those substitutes anymore. So we are now more inclined to eat American food than the regular home food that we used to. Not that if we found the home food we would not, but I am not going out of my way to look for them anymore.’ (in-depth interview)
Influence of age of migration	N/A	‘It's just that you know I think that because again I coming here as an adult that I was basically schooled in one way of preparing food and that is what I have just continued to do. I have not really changed my style of cooking. Maybe I changed.' (in-depth interview) ‘So I think that it sort of set it in stone what I would like to eat…Those things become much more important to me so that even going forward I always try to keep an element of that ethnic food and traditional you know kitchen cooking.’ (in-depth interview)	‘Well, when I came here. Actually when I came here I did not like the food, I didn't like the meat, it was too fatty for me. So as soon as I came, I was not eating that much so I lost a lot of weight, I was almost 90 lbs. But eventually I got used to, learn how to shop the meat that has very lean meat, bread that does, back home bread we make it at home, so the ones that I buy at the shop was very spongy, I didn't like them, so eventually I start learning about nutrition…So I was young then and it was the right time to come here and learn things, so maybe that influenced me I don't know. But from the beginning I was a healthy eater.' (in-depth interview) ‘Yes, coming to the States as an adult has influenced how I eat in the sense that what I, like I just explained to you, no matter all the food that they have in the shop, more than 80 % of those foods, I've never seen them, and the way they are prepared, the way they are cooked, they are never prepared the way what I'm used to, but over time, as I say, every day, and I meet more Nigerians…So they begin to show me where to get the items, where to get the recipes and how to make them. So I'm better off now than the time I came’ (in-depth interview)
Changes in environmental factors			
Variations in availability, quality and cost of cultural foods Poor quality and limited affordability for US-born Blacks Wide-scale availability for Caribbean foods, but not all, particularly fruit Varying availability and costliness for African foods	‘When you say availability for us to be African Americans and to be living in a certain area, you're also talking about quality of food. Like you said, it's expensive, it's not really what I want, and the meat is not fresh and sometimes the vegetables.’ (focus group) ‘You know, finances have a lot to do with where you shop. Star market, Shaws, Brothers…what's available, what can I get to in terms of transportation? And like you said, sometimes even saying ‘oh I got a deal.’ You buy a two-dollar bag of vegetables, and they still rotten or has a poor shelf life.’ (focus group) ‘When you say availability for us to be African Americans and to be living in a certain area, you're also talking about quality of food. Like you said, it's expensive, it's not really what I want, and the meat is not fresh and sometimes the vegetables.’ (focus group)	‘I can't say everything is available. But most things are.' (focus group) ‘Caribbean food is sold everywhere around Boston. You can find different places that sell different Caribbean food…And then, you'd find Chinese with something similar to the Trinidadian. You may find, you know, Vincentian, with their breads. It's on a different corner, but you can find cultural food for us.’ (focus group)	‘Yes, that's one other thing. Some of the vegetables we use I can't find here. For instance, to make the fish soup, we always have to use what they call bitterleaf. When I do find the bitterleaf here they are not fresh, they are either frozen or dried up…The spinach they have here is not the kind we have in Nigeria.' (in-depth interview) ‘There's a lot of things that we get, where that get imported. So there's a lot of Cape Verdean food that I could still have living in Boston because either my family members will bring it from Cape Verde or you can buy anything.' (in-depth interview) ‘It's expensive but it's available. There's (name of store) and they have all the foods from Ethiopia, but it's expensive, pricewise it's expensive.' (focus group)
Barriers of transportation, time constraints and work environments Caribbean-born and African-born Blacks expressed time as the primary barrier to eating cultural diets US-born participants indicate concerns with transportation and cost as a barrier African-born Blacks indicated cost as a barrier	‘A lot of people don't have transportation, access.’ (focus group) ‘Once again, which requires transportation. (Indecipherable) You can't bring nine bags on no bus.’ (focus group) ‘A lot of times you have to cherry pick, you know what I mean? You have to go the one store to get this, you go to another store to get that. You have to watch your budget.’ (focus group)	‘I say most of my time is on the weekend. I feel like I have a little bit more time. Because if you get home at five, who's gonna whip up a huge meal that's gonna take a couple hours? Unless you stay in your home on a Saturday, you can put in all the slow cooking thing in your house and do your laundry.' (focus group)	‘No, no, I'm saying now I have very little time to even look around for it anymore. So I'm just succumbing to whatever, for instance, Thanksgiving is coming. Every Thanksgiving, I prepare the usual American meal for my family because that's what they expect. I will throw in one or two Nigerian meals in the middle of it, but the main thing is still turkey and you know all those, with the trimmings’ (in-depth interview) ‘You don't have time to make that delicious food. We don't have time. We make it once a week, injera and something, otherwise we don't have no time. We are eager to eat when we see it.’ (focus group)

N/A, not applicable.

#### Satia-Abouta model of dietary acculturation topic area 1: changes in psychosocial factors and taste preferences

In comparison with US-born subjects, Caribbean/Latin American-born and African-born Blacks indicated a prominent place for a variety of culturally specific fruits, vegetables, fish and spices and seasonings in their diet. Spicy peppers and fresh herbs, for example, were commonly mentioned among Caribbean/Latin American-born and African-born Blacks. As one Caribbean-born participant described:

‘I would use more curry and turmeric, and I make my own seasoning like with onions and garlic and peppers. A mixture of sweet marjoram and all that kind of stuff together and make my own seasoning, so I would (not) use too much of the salty stuff.’ (focus group)

Meanwhile, US-born Blacks mentioned more fried foods, especially chicken, other meat such as pork, and specific vegetables and starches such as collard greens and sweet potatoes as being cultural foods. Rice was commonly mentioned among all three groups.

We found variations between the groups studied in the value and preference for maintaining a traditional diet compared with adopting a Western diet. Caribbean/Latin American-born and African-born Blacks expressed a strong preference for cultural foods, which they linked with their identity. As one East African-born participant described:

‘Well, I can't live without it. I have to have it, so, it's important because I grew up with it. Also, you just get used to it. I cannot not eat it at least for a couple of days. So, it has value, it's important, that's how I identify.' (in-depth interview)

US-born Blacks had a more mixed response to cultural foods preferences, with some US-born Blacks stating that culture had a major influence on their diet and others stating a minor influence. The age at migration among African-born and Caribbean/Latin American-born participants influenced their dietary habits, with cultural diets shaping taste preferences during both youth and adulthood. Those who migrated in adulthood indicated having a strong preference and knowledge for their cultural style of cooking and use of food ingredients. Opinions varied among those who migrated as young children, with some expressing an affinity to cultural foods and others indicating a greater influence of the Western diet.

As expressed by a Caribbean/Latin American-born participant who migrated to the USA as an adult:

‘So I think that it sort of set it in stone what I would like to eat…Those things become much more important to me so that even going forward I always try to keep an element of that ethnic food and traditional you know kitchen cooking.’ (in-depth interview)

Meanwhile, many of the US-born Blacks indicated a greater influence of a value for health on their eating habits. A theme that emerged from all three groups included the cultural and deeper meaning of food as symbolic of love and connection. Gatherings with family and friends, particularly around the holidays, were also noted as fostering adherence to cultural diets.

#### Satia-Abouta model of dietary acculturation topic area 2: changes in environmental factors

Poor quality and limited affordability and restaurant availability of cultural foods were noted concerns for US-born Blacks. Conversely, Caribbean/Latin American-born Blacks indicated wide availability of Caribbean foods, but expressed difficulty in finding some tropical fruits. As one Trinidadian participant explained:

‘Caribbean food is sold everywhere around Boston. You can find different places that sell different Caribbean food…And then, you'd find Chinese with something similar to the Trinidadian.’ (focus group)

Meanwhile, the African-born Black participants suggested varying availability, cost and quality of culturally appropriate foods. Both Caribbean/Latin American-born and African-born participants indicated finding similar foods and products at other local ethnic stores (i.e. Asian or Hispanic), given commonalities with other ethnic cuisines.

Time constraint was an environmental barrier to adherence to cultural diets among Caribbean/Latin American-born and African-born Blacks. To a smaller extent, the complaints from co-workers about the strong smell of cultural foods in the work environment presented as a barrier for consuming cultural diets among Caribbean/Latin American-born and African-born Blacks. Conversely, US-born participants indicated concerns with transportation and affordability as key barriers. As evidenced by one participant, carrying groceries on public transportation served as a barrier, as she explained, ‘You can't bring nine bags on no bus’ (focus group).

There was also a theme across groups which explored adaptive strategies in response to environmental barriers such as resource availability (time and food) and knowledge. The foreign-born participants expressed adaptations to when and how they cooked their meals as well as their preparation methods and portion sizes. For example, adaptive strategies included cooking in bulk on the weekends, using more easily accessible ingredients or canned versions of products instead of fresh options, and bringing food products from their home country when travelling. Conversely, US-born participants expressed changes in preparation and ingredients from their traditional ‘African American/soul food’ diet to healthier options for health reasons and a prioritisation of health.

## Discussion

In this qualitative study, use of Satia-Abouta's model^(20)^ of dietary acculturation appropriately framed the exploration of the influence of culture and ethnicity on the diet of US-born, African-born and Caribbean/Latin American-born Blacks living in Boston, MA, USA. Overall, the study findings underscore the complexity of the cultural diversity within and across the different ethnic groups of Blacks living in the USA and the consequent heterogeneity of the dietary experiences. These findings also validate the work of prominent nutrition researcher, Dr Shiriki Kumanyika, who proposed a research paradigm for working with Black communities. Arguing that to effectively address health disparities among Blacks in the USA there needs to be a greater focus on cultural and psychosocial processes, social and historical contexts and the obesogenic physical and economic environments^(26)^. Additionally, this study highlights the need to explore within-group differences and address the misconception of Blacks living in America as a homogeneous group^(26)^.

These study results identified cultural assets and their importance in influencing the diets of Caribbean/Latin American-born and African-born Blacks, who expressed a strong valuation of cultural food preferences as an important part of their identity. As some dietary recommendations encourage fruit and fish intake for chronic disease prevention^(27)^, the cultural preferences of these food groups, particularly among Caribbean/Latin American- and African-born Blacks, suggest an opportunity for promoting healthy culturally specific foods among Black immigrants. Meanwhile, although the traditional African American/‘soul food’ cuisine such as fried chicken, collard greens and macaroni and cheese were discussed among US-born Blacks, many participants discounted the influence of this cultural diet on their current dietary habits.

The geography and placement of the study in a northeastern US city also has important implications, particularly for the diet of US-born Blacks. Many of the US-born participants, for example, were a generation or two removed from their southern roots, with family members migrating from the south during the Great Migration in the early-to-mid 20th century. These results are thereby in alignment with the research leading to the development of the African American Acculturation Scale^(28,29)^. While acculturation is typically used to describe changes among immigrants after emigration, US-born African Americans likewise have undergone a form of acculturation by being on a continuum between reflecting a traditional cultural orientation and being immersed in African American culture to an acculturated orientation and low immersion in African American culture. In fact, for a small fraction of the US-born participants, there was allusion to a desire to clearly differentiate from their Black southern roots and identity, with a negative value label placed on soul food. Geography and environmental factors such as availability and affordability of culturally appropriate foods are therefore important factors in dietary behaviours among US-born Blacks, as well as the complex nature of African American identity formation in the racialised context of the USA^(30–32)^.

Results of this study also demonstrate the influence of psychosocial and environmental factors on the acculturation process among foreign-born Blacks. Themes differed somewhat by demographic factors such as age of migration and length of residence. For example, dietary changes with increased length of residency ranged from both unhealthy adaptation such as increased processed foods to healthy changes such as healthier adaptations to cultural foods. These findings are supported by recent research suggesting changes in diet with increased length of residency among Black immigrant groups, particularly increases in polyunsaturated fats and red/processed meat after living in the USA for more than 10 years^(33)^.

In terms of environmental influencers, a major theme was the widespread availability of cultural foods in both restaurants and grocery store outlets among foreign-born study participants, with the ethnic diversity and prevalence of ethnic enclaves in Boston potentially contributing to this availability. According to a 2015 report, 32·8 % of the Massachusetts Black population is foreign-born, with Boston including the largest Black community^(34)^. Based on sociology research, ethnic enclaves may foster less acculturation, reinforce norms and health behaviours, and contribute to resource sharing based on social networks, which ultimately facilitates adherence to culturally appropriate diets^(35)^. Additionally, when cultural foods were not available, participants utilised adaptive strategies to adhere to their diets, such as using different ingredients, or seeking grocery store outlets of different ethnic groups with similar foods (i.e. Chinese or Arab markets). For East African-born study participants, for example, revisions were made to recipes for *injera* bread based on grains available and affordable in the Boston community. While culturally appropriate foods were noted as available, African-born participants noted affordability as a concern and an influence on changes in their diets. The fast-paced ‘American lifestyle’ of the USA and consequent time constraints were a consistent challenge for all study participants, as is the case for Americans generally^(36)^, with some adaptive strategies such as cooking in bulk during the weekends.

While income and education are noted in the literature as influencing dietary purchases and related lifestyle behaviours, these themes did not differ among foreign-born Blacks in our study. One possible explanation is the application of intersectionality theory^(1,2)^, which suggests the non-additive effects of social identities such as sex/gender, race/ethnicity, class, immigration status, etc. and as it relates to the study of behaviour, health and disease, how these different intersections of identity contribute or protect against health inequalities^(37,38)^. In this case, the potential influence of education on health could be muted by the intersection and non-additive effects of social identities beyond being college educated. Additionally, while there has been a focus on culture and acculturation in describing immigrant health, some researchers note the importance of considering the socio-historical contexts and the different social experiences of diverse immigrant groups^(38)^. It is possible that the intersectionality of these sociodemographic factors might differentially shape the acculturation process among Black immigrant groups in comparison with other immigrant groups. This qualitative study raises questions about the potential for this effect, which may be explored in future studies.

While this is one of the first studies in the USA to qualitatively apply the dietary acculturation model to the US Black population, there are limitations worth noting. Firstly, due to challenges in recruitment, we only conducted one focus group for the African-born ethnic group. Additionally, the African participants were predominantly from West African (i.e. Nigeria, Cape Verde) and Eastern African countries (Ethiopia, Somalia) and not representative of countries throughout the continent. Although we collaborated with a community-based organisation whose main clients were African immigrants, logistical concerns and leadership transitions within the organisation presented a roadblock for facilitating an additional focus group. Time constraints also posed a concern for many participants who expressed initial interest, but could not commit to full participation. Future studies could recruit more study participants and consider additional incentives and strategies to address the issue of time constraints in this demographic (i.e. providing transportation, childcare services, etc.). During the recruitment process, many of those approached expressed time constraints as a barrier for participation, which potentially introduced selection bias. Additionally, in our analyses we did not account for potential cultural differences of study participants who identify as Black racially but who are also ethnically Hispanic (i.e. participants from Honduras, Puerto Rico, etc.). Future studies could therefore explore these potential influences. Additionally, this study was restricted to those households without children in order to look specifically at the effects of culture on diet without the influence of children's dietary preferences and needs. However, since many households have children, future studies could examine how their presence may influence the study results.

Given the geographic limitation of this research, future studies should examine Blacks in other cities throughout the USA. The cultural influences on diet among US-born Blacks in the South, for example, may differ from US-born Blacks in other parts of the USA. Additionally, as mentioned above, Boston includes an ethnically diverse Black population, and the formation of ethnic enclaves has contributed to a demand for cultural foods. The dietary acculturation process and adaptive strategies may vary across region, however. Additionally, with the growing proportion of second- and third-generation Caribbean/Latin American-born and African-born people (i.e. children and grandchildren of immigrants), future studies could explore the impact of biculturism on dietary preferences and diet-related disease risk among this demographic. The application of intersectionality theory among Black immigrants as it relates to dietary and other lifestyle behaviours is also worthy of further exploration. While most of the literature exploring acculturation has focused on Asian and Hispanic populations^(15,16,18)^, more literature is needed to understand the Black immigrant experience with the consideration of the differences in the US sociopolitical and historical contexts and racialisation of these groups.

Overall, this study suggests the importance of considering the ethnic diversity in the US Black population in nutrition science and research. With the growing Black immigrant population in the USA, these research findings will be important for the evolving field of nutrition. Place of birth and cultural origin among the US Black population, and acculturation among Black immigrants have implications for a range of nutrition research studies, from interventions to nutrition epidemiology. For example, intervention studies targeting African Americans and Black immigrants should be culturally tailored and integrate the culturally influenced aspects of diet. This study also informs possible differences in diet that might underlie diet-related diseases among the US Black population having important implications for future nutrition and health disparities research.

### Conclusions

This novel study illustrates the complexity of the Black dietary experience and the influence of culture and ethnicity on the diet of an ethnically diverse Black population living in Boston, MA, USA. As outlined in the model of dietary acculturation, changes in environmental and psychosocial factors contribute to dietary changes among Blacks who migrate to the USA and consequently the dietary diversity in the US Black population. This influence of the diversity in culture among Blacks should continue to be explored in qualitative and quantitative studies to inform nutrition science from nutrition intervention research to diet-related health disparities research.
